# Human SLURP-1 and SLURP-2 Proteins Acting on Nicotinic Acetylcholine Receptors Reduce Proliferation of Human Colorectal Adenocarcinoma HT-29 Cells

**Published:** 2014

**Authors:** E. N. Lyukmanova, M. A. Shulepko, M. L. Bychkov, Z. O. Shenkarev, A. S. Paramonov, A. O. Chugunov, A. S. Arseniev, D. A. Dolgikh, M. P. Kirpichnikov

**Affiliations:** Shemyakin and Ovchinnikov Institute of Bioorganic Chemistry, Russian Academy of Sciences, Miklukho-Maklaya str., 16/10, Moscow, 117997, Russian Federation; Biological Department, Lomonosov Moscow State University, Vorobievy Gory, 1, Moscow, 119991, Russia; Moscow Institute of Physics and Technology (State University), Institutskii per., 9, Dolgoprudny, Moscow Region, 141700, Russia

**Keywords:** nicotinic acetylcholine receptor, bacterial expression, refolding, Lynx, colon cancer

## Abstract

Human secreted Ly-6/uPAR related proteins (SLURP-1 and SLURP-2) are produced by various cells, including the epithelium and immune system. These proteins act as autocrine/paracrine hormones regulating the growth and differentiation of keratinocytes and are also involved in the control of inflammation and malignant cell transformation. These effects are assumed to be mediated by the interactions of SLURP-1 and SLURP-2 with the α7 and α3β2 subtypes of nicotinic acetylcholine receptors (nAChRs), respectively. Available knowledge about the molecular mechanism underling the SLURP-1 and SLURP-2 effects is very limited. SLURP-2 remains one of the most poorly studied proteins of the Ly-6/uPAR family. In this study, we designed for the first time a bacterial system for SLURP-2 expression and a protocol for refolding of the protein from cytoplasmic inclusion bodies. Milligram quantities of recombinant SLURP-2 and its 13C-15N-labeled analog were obtained. The recombinant protein was characterized by NMR spectroscopy, and a structural model was developed. A comparative study of the SLURP-1 and SLURP-2 effects on the epithelial cell growth was conducted using human colorectal adenocarcinoma HT-29 cells, which express only α7-nAChRs. A pronounced antiproliferative effect of both proteins was observed. Incubation of cells with 1 μM SLURP-1 and 1 μM SLURP-2 during 48 h led to a reduction in the cell number down to ~ 54 and 63% relative to the control, respectively. Fluorescent microscopy did not reveal either apoptotic or necrotic cell death. An analysis of the dose-response curve revealed the concentration-dependent mode of the SLURP-1 and SLURP-2 action with EC50 ~ 0.1 and 0.2 nM, respectively. These findings suggest that the α7-nAChR is the main receptor responsible for the antiproliferative effect of SLURP proteins in epithelial cells.

## INTRODUCTION

**Fig. 1:**
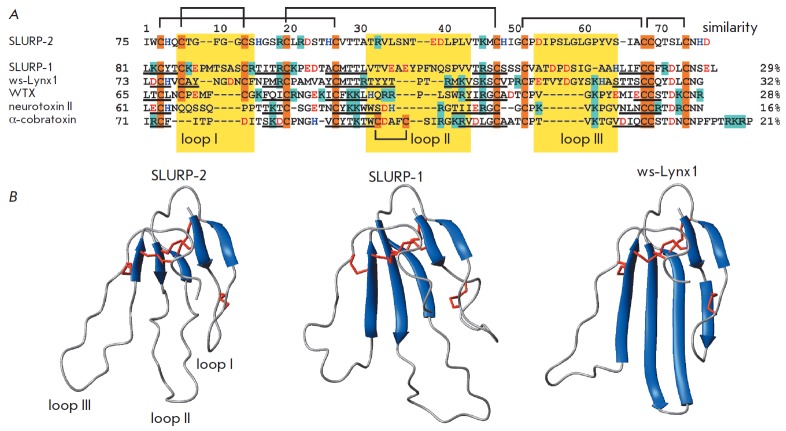
Comparison of the structures of three-finger proteins of the Ly-6/uPAR family. (A). Amino acid sequence alignment of human SLURP-1, SLURP-2, and a water-soluble domain of human Lynx1 (ws-Lynx1), the WTX neurotoxin from* Naja kaouthia*, the neurotoxin II from *Naja oxiana*, and the α-cobratoxin from *Naja kaouthia. *The positively charged (Arg/Lys), negatively charged (Asp/Glu), His and Cys residues are highlighted by color, the disulfide bonds are shown. The protein fragments forming β-strands are underlined. The loop regions are highlighted by a yellow background. The amino acid sequence homology between SLURP-2 and other three-finger proteins was calculated using the CLUSTAL W2 software. (B). Comparison of the SLURP-2 structure model with the spatial structures of SLURP-1 (Shenkarev* et. al*, submitted, PDB 2MUO) and ws-Lynx1 ([23], PDB 2L03)

The nicotinic acetylcholine receptor (nAChR) is a ligand- gated ion channel found both in the central and peripheral nervous systems and in many other human tissues, including the epithelium [1, 2]. During last decade, in higher animals, proteins were found that belong to the Ly-6/uPAR family and have a modulating effect on nAChRs (Lynx1, Lynx2, Lypd6, SLURP-1, and SLURP-2) [3-7]. A conserved location of Cys residues (*Fig. 1*), forming disulfide bonds, indicates the homology of the spatial structure of the Lynx and SLURP proteins to the three-finger structure of snake venom α-neurotoxins, which are highly efficient and specific nAChR inhibitors [8].

SLURP-1 and SLURP-2 are secreted proteins found in many human tissues, including the epithelium, as well as the immune and nervous systems [5, 6, 9, 10]. SLURPs affect the growth, migration, and differentiation of epithelial cells, and they are involved in the control of inflammation and tumors [6, 11, 12]. With the Het1A keratinocyte line, it was demonstrated that SLUR P-1 exhibits antiproliferative activity and promotes apoptotic cell death [11], while SLURP-2 accelerates keratinocyte growth, delaying their differentiation and reducing the response to proapoptotic signals [6]. In addition, SLURPs regulate wound healing in the skin and mucous membranes [13] and are involved in the protection of skin cells from the oncogenic transformation caused by nitrosamines (nicotine derivatives) [14, 15]. Probably, SLUR P-1 and SLUR P-2 act as auto/ paracrine regulators and their effects are mediated by the interaction with the nAChRs presented on the cell membrane surface of keratinocytes and immune cells [10, 16] . α7 and α3β2 nAChRs are the putative targets for SLUR P-1 and SLUR P-2, respectively [6, 11]. Recently, SLURP-1 expression was detected in HT-29 human colorectal adenocarcinoma cells and the level of endogenous production of SLURP-1 in these cells was demonstrated to decrease significantly when the cells are treated with nicotine [17]. At the same time, HT-29 cells were shown to be able to express only the α7 type of nAChRs [18].

Currently, the structural and functional properties of human SLURP-1 and SLURP-2, as well as their mechanism of action, remain poorly studied. The main stumbling stones in studying SLURP-1 and SLURP-2 are related to the inability to produce adequate amounts of protein samples from natural sources, as well as to the problems of recombinant production of these proteins with a native sequence and spatial structure. As a consequence, the majority of previously published results were obtained using hybrid constructs containing, along with the SLURP protein sequence, additional polypeptide sequences that can significantly affect resultant activity. For example, in the study [6], a construct fused with the SUMO protein (the total protein weight was 22 kDa, of which only ~ 8 kDa accounted for SLURP-2) was used to investigate SLURP-2.

In the present study, an efficient bacterial system for human SLURP-2 production in the form of cytoplasmic inclusion bodies was developed for the first time and protein refolding protocol was proposed. The resulting recombinant analog differs from the wild type protein by the presence of one additional residue (N-terminal Met). A high-expression yield (~ 5 mg of the refolded protein per 1 L of the bacterial culture) enabled production of milligram quantities of the recombinant protein and its ^13^C^15^N-labeled analog. The development of this system of recombinant production opens up new perspectives for structural and functional studies of SLURP-2. For example, in this paper, we demonstrated for the first time a significant antiproliferative effect of SLURP-1 and SLURP-2 on HT-29 line cells. This suggests that α7-nAChR plays the main role in transduction of SLURP-induced signals resulting in the reduction of growth of epithelial cells.

## EXPERIMENTAL

**Cloning and bacterial production of SLURP-2.**

The *slurp-2 *gene encoding 75 amino acid residues of the human SLURP-2 protein (*Fig. 1A*) was constructed from overlapping synthetic oligonucleotides using PCR and with allowance for the codon frequency in* E. coli *(Evrogen, Moscow, Russia). The *slurp-2 *gene was cloned into the *pET-22b(+) *expression vector (Novagen) at NdeI and BamHI restriction sites. BL21 (DE3) *E. coli *cells transformed with the *pET-22b(+)/ slurp-2 *vector were cultured at 37°C on a TB medium (12 g of bacto-tryptone, 24 g of yeast extract, 4 mL of glycerol, 2.3 g of KH_2_PO_4_, 12.5 g of K_2_HPO_4_ per 1 L of the medium, pH 7.4) in a Bioflo 3000 fermentor (New Brunswick Scientific) under automatically maintained conditions of a relative oxygen content in the system of not less than 30% of the maximum achievable value. The *slurp-2 *gene expression was induced by adding isopropyl β-D-1-thiogalactopyranoside (IPTG) to the final concentration of 0.05 mM at an optical density of the cell culture of 1.0 OD. After the induction, the cells were cultured for 8 h.

In order to produce the 13C-15N-labeled SLURP-2 analogue, 1 L of the cell culture, which had been preliminarily grown on a TB medium in flasks to a cell density of 1.0 OD, was centrifuged at 1,000 g for 20 min. The cell pellet was aseptically re-suspended in 1 L of the M9 minimal medium (6 g of Na_2_HPO_4_, 3 g of KH_2_PO_4_, 0.5 g NaCl, 2 g of NH4Cl, 240 mg of anhydrous MgSO_4_, 11 mg of CaCl_2_, 3 g of glucose, 2 mg of yeast extract, 200 μL of 5% thiamine chloride per 1 L of the medium, pH 7.4) containing 13C-glucose and ^15^N-NH_4_Cl (CIL) as a source of glucose and nitrogen, respectively. Induction and further growth were carried out similarly to growth on the TB medium.

**Purification and refolding of recombinant SLURP-2.**

Inclusion bodies containing SLURP-2 were isolated and washed according to the protocols described previously for SLURP-1 [19]. The washed inclusion bodies were re-suspended in 30 mM Tris-HCl, pH 8.7, containing 8 M urea, 0.4 M sodium sulfite, 0.15 M sodium tetrathionate in the amount of 10 mL of the buffer per 1 g of inclusion bodies. The suspension was disintegrated by ultrasound (Branson Digital Sonifier) at an output power of 50 W and 4 °C for 1 min and left for 8 h under mild stirring. The suspension was then centrifuged at 36,000 g, 4 °C, for 30 min, and the supernatant was diluted 10 times with 2 M urea. Afterwards, the sulfited SLURP-2 sample was loaded onto a column of DEAP-sferonit- OH (joint development by the Institute of Highly Pure Biopreparations, St. Petersburg, and the Institute of Bioorganic Chemistry, Moscow, Russia) preliminarily equilibrated with the buffer A (30 mM Tris-HCl, pH 8.0). After loading of the protein, the column was sequentially washed with the buffer A, with the buffer A supplemented with 1 M NaCl, and with the buffer A supplemented with 8 M urea. The sulfited SLURP-2 was eluted with the buffer A supplemented with 8 M urea and 0.5 M NaCl. Fractions containing SLURP-2 were added with a 1,000-fold (relative to the protein) molar excess of DTT. Reduced SLURP-2 was purified by HPLC (Jupiter C4, A300, 10×250 mm, Phenomenex). SLURP-2 was eluted with the acetonitrile gradient (20 – 45%) in the presence of 0.1% TFA for 40 min. The resulting reduced SLURP-2 sample was lyophilized and dissolved in the refolding buffer containing 50 mM Tris-HCl, pH 9.0, 2 M urea, 0.5 M L-arginine, 2 mM GSH, and 2 mM GSSG to a final protein concentration of 0.1 mg/mL. Refolding was performed at 4 °C for three days. Analysis and purification of SLURP-2 after refolding was performed by HPLC (Jupiter C4, A300, 4.6x250 mm, Phenomenex). The resulting refolded SLURP-2 sample was lyophilized.

**NMR spectroscopy and modeling of the structure of SLURP-2.**

NMR spectra of ^13^C-^15^N-labeled and unlabelled SLURP-2 (sample concentration was 0.5 mM) were acquired on an AVANCE-700 spectrometer (Bruker) at 30 °C.

To model the structure of SLURP-2, the ws-Lynx1 protein (PDB 2L03) was used as a template. Alignment of amino acid sequences was performed on the Clustal web server (www.clustal.org). The model was constructed using the Modeller V8.2 software [20].

**Working with the HT-29 cell line.**

HT-29 colorectal adenocarcinoma cells (Institute of Cytology of the Russian Academy of Sciences, St. Petersburg, Russia) were maintained in a RPMI-1640 medium (PanEco, Moscow, Russia) supplemented with 5% fetal bovine serum (Hyclone, Thermo Fisher Scientific). The cells were maintained in a humidified atmosphere (37 °C, 5% CO_2_) and were passaged every 48 h. Sixteen hours before the experiment, the cells were seeded in 96-well culture plates at a density of 10^4^ cells per well. After adsorption of cells, they were added with SLURP-1 and SLURP-2 samples (the recombinant SLURP-1 sample was prepared according to the protocol described in [19]). All sample dilutions were performed in the culture medium. The cells were incubated with SLURP-1 and SLURP-2 samples for 48 h. Cell proliferation was studied using a WST-1 reagent (water soluble tetrazolium salt 1, Santa Cruz). WST-1 was dissolved in 20 mM HEPES (pH 7.4), and an electron transport reagent, 1-m-PMS (1-methoxy- 5-methylphenazinium methyl sulfate, Santa Cruz), was dissolved in deionized water, after which the solutions were mixed and added to plate wells in the amount of 0.5 mM WST-1 and 20 μM 1-m-PMS per well. After 3 h incubation with WST-1, cell viability was evaluated spectrophotometrically by absorbance at 450 nm with alignment of the background at 655 nm (BioRad Spectrophotometer 680, BioRad Laboratories).

**Fluorescence microscopy.**

To study the morphology of tumor cell nuclei, the Hoechst 33342 dye (Sigma) was used. Necrotic cell death was determined by staining the cells with propidium iodide (Sigma). The cells were treated in the same manner as for the proliferation assay, but after incubation with SLURP-1 and SLURP-2 samples, the cells were stained with 1 μM Hoechst 33342 dye and 0.5 μM propidium iodide, and then the nuclei were analyzed using a Nikon Eclipse TS100-f microscope (Nikon Corp) with a 40x lens.

## RESULTS AND DISCUSSION

**Bacterial production and refolding of SLURP-2.**

Previously, using the example of the weak toxin WTX from *Naja kaouthia *venom [21], ws-Lynx1 modulating the nAChR activity [22], and the human SLURP-1 [19], it was demonstrated that the optimal way for the production of recombinant three-finger containing the fifth disulfide bond in the first loop (*Fig. 1A*) is production in the form of cytoplasmic inclusion bodies, followed by refolding. This approach was also used for the recombinant production of SLURP-2. The yield of the SLURP-2 with reduced disulfide bonds was approximately 40 mg and 15 mg per 1 L of the bacterial culture on rich (TB) and minimal (M9) media, respectively. However, the refolding protocols developed earlier for other three-finger proteins [19, 21, 22] were ineffective for the refolding of SLURP-2.

**Fig. 2:**
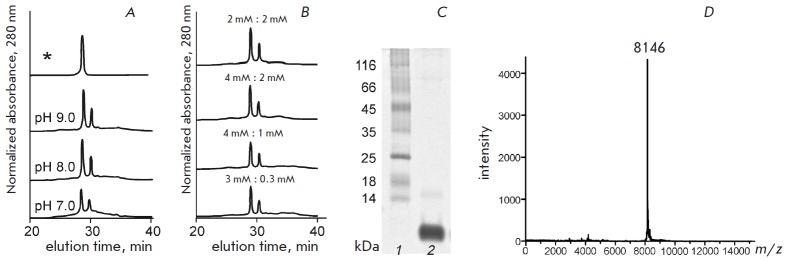
Analysis of recombinant SLURP-2. (A–B). The SLURP-2 refolding efficiency depends on the pH of the refolding buffer (A) and the concentration of the reduced (GSH) and oxidized (GSSG) forms of glutathione (B). The peak corresponding to the refolded SLURP-2 is denoted by an asterisk. (C). SDS-PAGE analysis of the refolded SLURP-2 after purification by HPLC. (D). Mass-spectrum of the refolded SLURP-2

To optimize conditions for the refolding of SLURP-2, different pH values (7.0–9.0) of the refolding buffer and different concentrations of the reduced (GSH) and oxidized (GSSG) forms of glutathione (4:1 mM, 4:2 mM, 2:2 mM, and 3:0.3 mM) were tested (*Figs. 2A and B*). Under the optimum conditions (see Experimental section), the yield of refolded SLURP-2 and ^13^C-^15^N-labeled analog was 4.6 and 3 mg per 1 L of the bacterial culture, respectively. The homogeneity of the refolded SLURP-2 was confirmed by SDS electrophoresis (*Fig. 2C*), HPLC (*Fig. 2A*) and mass spectrometry (*Fig. 2D*). The molecular weight of the recombinant protein was 8146 Da, which, with allowance for the experimental error, was in accordance with the predicted weight of SLURP-2 (8145 Da) with five closed disulfide bonds and an additional N-terminal methionine residue. The formation of disulfide bonds was also confirmed by the Ellman’s reagent.

**NMR spectra and modeling of the structure of SLURP-2.**

**Fig. 3:**
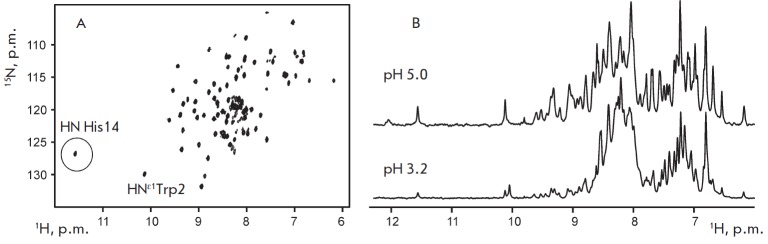
NMR analysis of recombinant SLURP-2. (A). 2D ^1^H-^15^N HSQC spectrum of 0.5 mM ^13^C-^15^N-labeled SLURP-2 (30 °C, pH 5.0). (B). Fragments of 1D ^1^H spectra of unlabeled SLURP-2 at pH 5.0 and 3.2

An analysis of the 2D ^1^H-^15^N correlation NMR spectrum of recombinant SLURP-2 (*Fig. 3A*) confirmed the homogeneity and purity of the obtained sample. A considerable dispersion of the ^1^H^N^ backbone signals (7 to 9.7 ppm) indicated the presence of β-structural regions in the protein. A single set of signals was observed in the NMR spectra, which indicated a lack of conformational heterogeneity due to *cis-trans *isomerization of Xxx- Pro peptide bonds. In this aspect, the SLURP-2 protein is similar to ws-Lynx1, which has a single structural form in the solution [23], and differs from SLUR P-1, which in the solution forms two equally populated structural states due to slow (on the NMR timescale) isomerization of the Tyr39-Pro40 peptide bond [19]. In this regard, it should be noted that, according to the amino acid sequence analysis, SLURP-2 has greater homology with ws-Lynx1 than with the SLURP-1 (32 and 29%, respectively, *Fig. 1A*). Interestingly, human Lynx1 and SLURP-2 proteins are the products of a single gene located on the chromosome 8 that arise during alternative splicing.

The similarity of the SLURP-2 and ws-Lynx1 structures is also indicated by the presence of the characteristic downfield ^1^H^N^ signal at 11.6 ppm (circled, *Fig. 3A*). According to the published spatial ws-Lynx1 structure [23], a significant downfield shift of the ^1^H^N^ Asn15 signal is caused by the formation of a hydrogen bond with a side chain of His4. A similar hydrogen bond in the structure of SLURP-2 can be formed between the side chain of His4 and the backbone amide group of His14. A reduction in the pH value of the SLURP-2 sample from 5 to 3 resulted in a significant decrease in the signal intensity in the downfield region (8.7–9.7 ppm) of the 1H NMR spectrum with a simultaneous increase in the signal intensity around 8 ppm (*Fig. 3B*). This indicated partial disruption of the spatial structure of the protein, accompanied by transitions of individual fragments from the β-structural conformation to the random coil conformation. Similar pH-induced denaturation was previously observed for ws-Lynx1 but not for SLURP-1 (Shenkarev et al., unpublished data).

Taking into account this indirect evidence of the spatial structure similarity, a model of SLURP-2 was built (*Fig. 1B*) based on the known structure of the ws- Lynx1 protein. The resulting model shows the typical three-finger fold with β-structural core enclosing two antiparallel β-sheets formed by five β-strands.

**Effects of SLURP-1 and SLURP-2 on the HT-29 cells**

**Fig. 4:**
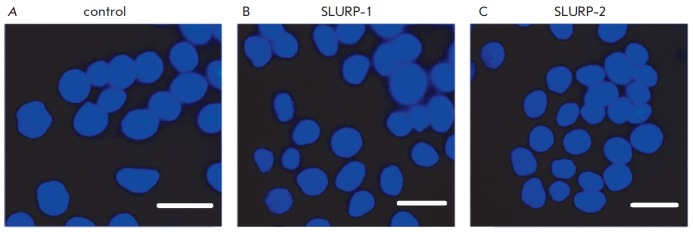
Effect of SLURP-1 and SLURP-2 on the morphology of colorectal adenocarcinoma HT-29 cell nuclei. (A). Cells without SLURP proteins. (B–). Cells after 48 h incubation with 1 μM SLURP-1 and 1 μM SLURP-2, respectively. Cell nuclei were stained with Hoechst 33342 and propidium iodide. Bar scale is 10 microns

Incubation of colorectal adenocarcinoma HT-29 cells with the SLURP-1 and SLURP-2 at a concentration of 1 μM for 48 h resulted in a significant decrease in the cell number to 54 ± 2% and 63 ± 2% relative to the control, respectively. An analysis of cell nuclei morphology by fluorescence microscopy demonstrated that neither SLURP-1 nor SLURP-2 causes apoptotic or necrotic death of HT-29 cells (*Fig. 4*). For example, a reduction in the cell density was not accompanied by a change in the morphology of most of the cell nuclei compared to the control, and staining of cells with propidium iodide showed no increase in the fraction of necrotic cells (3 ± 1% in the control and in wells containing 1 μM SLUR P-1 and SLUR P-2). Therefore, the observed effects of SLURP-1 and SLURP-2 are associated with inhibition of HT-29 cell’s proliferation.

**Fig. 5:**
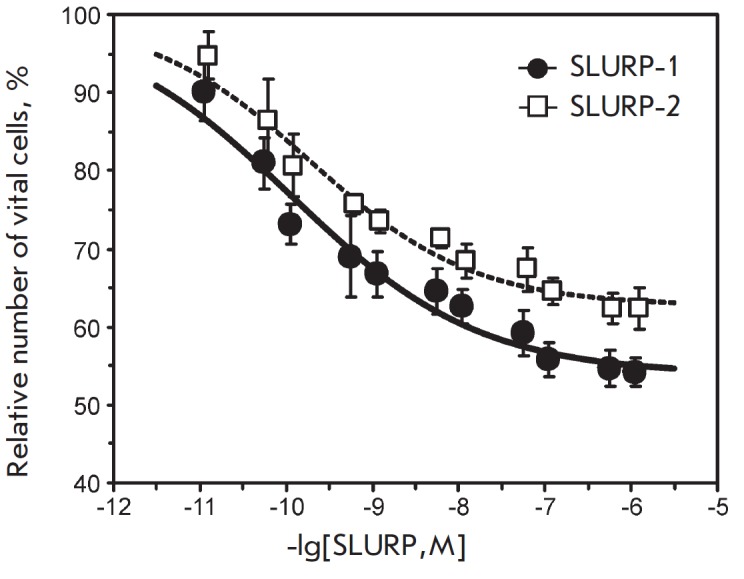
Effect of SLURP-1 and SLURP-2 on the growth of colorectal adenocarcinoma HT-29 cells as determined by the WST-1 test. Each point is the mean ± S.E. of 3 independent experiments. Dose-effect curves of SLURP-1 and SLURP-2 (the percentage ratio of viable cells to the control) were fitted to the Hill equation (y=A1+(100%-A1)/ (1+([SLURP]/EC_50_)^nH^). The calculated parameters EC_50_, nH, and A1 were 0.11 ± 0.05 nM, 0.4 ± 0.1, and 54 ± 2%, respectively, for SLURP-1, and 0.19 ± 0.07 nM, 0.5 ± 0.1, and 63 ± 2%, respectively, for SLURP-2

A comparative analysis of results of WST-1 test revealed that SLURP-1 and SLURP-2 significantly inhibit the growth of HT-29 tumor cells. An analysis of the dose-effect curve showed that the inhibitory effect of SLUR P-1 and SLUR P-2 depends on their concentration (*Fig. 5*). The half maximal effective concentration (EC_50_) was ~ 0.1 nM for SLURP-1 and ~ 0.2 nM for SLURP-2. The maximum inhibitory effect was achieved at a protein concentration of about 1 μM (*Fig. 5*).

HT-29 cells were demonstrated to contain mRNAs encoding only α4, α5, α7 and β1 subunits of the nAChR [18]. Due to the fact that only α7 subunits of this set can form functional receptors [1], the authors suggested that the α7-nAChR is the only nicotinic acetylcholine receptor presented in HT-29 cells [18]. Perhaps, this is the receptor that is involved in the regulation of interleukin- 8 release by HT-29 cells exposed to nicotine [18]. Based on these data, we may assume that α7-nAChR is the target of SLURP-1 and SLURP-2 proteins in HT- 29 cells.

between ^3^H-nicotine and ^3^H-epibatidine in Het1A keratinocytes, which, contrary to HT-29 cells, express different types of nAChR [24], it was suggested that the target of SLURP-1 is α7-nAChR and that SLURP-2 affects primarily α3β2-nAChR [6, 11]. In this case, SLURP-1 reduced the proliferation of keratinocytes [11], while SLURP-2, instead, increased it [6]. Therefore, it may be assumed that the inhibitory effect of SLURP-1 and SLURP-2 observed on Het1A and HT-29 cells is mediated by the interaction with α7-nAChR. In this case, the activating effect of SLURP-2 observed on keratinocytes could be due to the interaction with α3β2-nAChR. The lower antiproliferative activity of SLURP-2 compared to that of SLUR P-1 on HT-29 cells is probably associated with lower affinity of this protein to the α7-nAChR.

## CONCLUSIONS

In this study, an efficient system for the production of the human SLUR P-2 was developed for the first time and milligram quantities of the recombinant protein and its ^13^C-^15^N-labeled analogue were obtained. Recombinant SLURP-2 differs from the wild type protein by the presence of an additional N-terminal Met residue. The development of this system opens up new opportunities for structure-function studies of SLURP-2, including by site-directed mutagenesis. The antiproliferative effect of the SLURP-1 and SLURP-2 on the human colorectal adenocarcinoma HT-29 cell line has been described for the first time, and this effect is assumed to be mediated by the interaction with α7-nAChR. These findings provide a fresh look at the role of the nicotinic acetylcholine receptor and its distinct subtypes in the regulation of epithelial cell growth.

## Abbreviations

nAChR: nicotinic acetylcholine receptor
SLURP: secreted Ly-6/uPAR related protein
ws-Lynx1: water-soluble domain of human Lynx1
